# Timing of congenital cytomegalovirus diagnosis and missed opportunities

**DOI:** 10.3389/fped.2025.1475121

**Published:** 2025-02-04

**Authors:** Mallory R. Baker, Xing Wang, Ann J. Melvin

**Affiliations:** ^1^ Department of Audiology, Seattle Children’s Hospital, Seattle, WA, United States; ^2^ Biostatistics Epidemiology and Analytics in Research (BEAR), Seattle Children’s Research Institute, Seattle, WA, United States; ^3^ Department of Pediatrics, Division of Pediatric Infectious Disease, University of Washington and Seattle Children’s Research Institute, Seattle, WA, United States

**Keywords:** congenital cytomegalovirus (cCMV) infection, hearing loss, newborn screening (NBS), diagnostic delay, congenital infection, early intervention

## Abstract

**Objective:**

Although congenital cytomegalovirus (cCMV) is the most common congenital infection world-wide, many infected infants are not diagnosed at birth. Anticipating that infants with cCMV who are not tested at birth risk a delayed diagnosis, this study was conducted to investigate the timing of diagnosis for infants with cCMV and to determine the reasons for and impact of late diagnoses.

**Methods:**

Clinical, imaging and laboratory data, hearing and developmental outcomes were abstracted from medical records between 2009 and 2021 for infants with virologically confirmed cCMV.

**Results:**

One hundred and twelve children with confirmed cCMV were identified. Diagnosis was within the first three weeks of life for 60 (54%) (early diagnosis group/EDG) and after this time for 52 (46%) (late diagnosis group/LDG). Infants in the LDG were diagnosed via CMV PCR on neonatal dried blood spot specimens with the majority (71%) tested after identification of sensorineural hearing loss (SNHL). The median time to first CMV testing in the LDG was 12 (IQR 3–42) months. Symptoms consistent with cCMV were present at birth in 17 (33%) of the infants with delayed diagnosis. More infants in the EDG received antiviral treatment (*n* = 41, 68% vs. *n* = 19, 23%). Developmental outcomes were similar between groups. Applying different screening strategies to the total cohort, 100%, 66% and 92% could have been diagnosed at birth with universal, hearing targeted and expanded testing strategies respectively.

**Conclusion:**

The lack of formal protocols for cCMV testing leads to delayed diagnoses for many infants. This delay results in missed opportunities for monitoring, intervention, and treatment.

## 1 Introduction

Cytomegalovirus (CMV) transmission occurs in 0.5%–1% of all live births (1–4), making CMV infection the most common congenital viral infection world-wide. Signs and symptoms are apparent at birth in only 10%–15% of infants with congenital CMV (cCMV) manifesting along a spectrum from mild illness to severe disseminated multiorgan system disease (5). Long-term neurodevelopmental sequelae are more common in symptomatic infants but can also be seen in infants who appear to be asymptomatic at birth (1, 6, 7).

cCMV is the leading non-genetic cause of sensorineural hearing loss (SNHL) in children, accounting for almost 25% of hearing loss in children by age 4 years (8). cCMV-related SNHL, which can present at birth or develop later in childhood, occurs in 30%–70% of symptomatic and 10%–15% of asymptomatic at birth infants (9–14).

Early suspicion of cCMV is essential to assure a timely diagnosis, as viral isolation beyond 3 weeks of age may represent an acquired infection (15, 16). Infants older than 3 weeks of age can be diagnosed retrospectively by testing the neonatal dried blood spot (DBS) for CMV (17). However, by the time a cCMV diagnosis is entertained, the DBS may no longer be available or opportunities for early intervention are missed.

Recently several states have adopted some form of required education and/or screening for cCMV, however, no consistent recommendation for newborn CMV screening has been established across the country (18). Testing for cCMV is usually at the discretion of clinicians, which has been shown to lead to underdiagnosis (19, 20).

In 2008, the Seattle Children's Hospital (SCH) otolaryngology and audiology clinics started testing DBSs for CMV as part of the etiology work-up for SNHL. Subsequent clinical experience revealed missed diagnoses of cCMV. To better understand why these infants with cCMV failed to be diagnosed at birth, we conducted a retrospective cohort study of infants and children diagnosed with cCMV who entered the SCH medical system. We hypothesized that once an infant discharges from the birth hospital, diagnosis would be delayed and lead to missed opportunities for antiviral treatment and early developmental and audiologic intervention.

## 2 Methods

### 2.1 Patient population

We conducted a single-center retrospective study to identify the timing of diagnosis for infants with cCMV seen at SCH between 2009 and 2021. cCMV infection was defined as documentation of a positive urine culture, urine PCR or blood PCR within 21 days of life; a positive saliva PCR followed by confirmatory urine culture, urine PCR or blood PCR within 21 days of life; or for those tested after 21 days of life, a positive CMV PCR from a neonatal DBS sample. The SCH Clinical Data Repository (CDR) was searched by International Classification of Diseases (ICD) diagnosis codes versions 9 and 10 and lab codes (Supplementary Table S1). The CDR contains electronic health record (EHR) data from all patients seen at SCH from 2009 onward. Patients with a birth date of 2004 and higher, with visit records between 2009 and 2021 were included. Children identified only by the general CMV ICD codes (078.5, B25.9) were excluded if alternative diagnoses were the reason for CMV testing (e.g., transplant, malignancy). The remaining infants underwent further clinical review to verify cCMV infection. Infants and children for whom a virologic diagnosis of cCMV was unable to be confirmed or for whom limited clinical data beyond the listed diagnosis was available were excluded (Supplementary Figure S1).

Demographic characteristics, birth history, newborn hearing screen, clinical presentation, antiviral treatment, audiologic results, developmental data, and laboratory and imaging evaluations were extracted from the EHR into a REDCap database (21). Initial data abstraction was done by AJM (Pediatric Infectious Disease), MRB (Pediatric Audiology) with assistance from AA and JK (University of Washington medical students). Data validation was done via duplicate review by AJM and MRB. Infants symptomatic at birth were defined as presenting at birth with one or more characteristic consistent with cCMV including: small for gestational age (SGA) (<10%), microcephaly (head circumference <3%), abnormal physical exam (generalized petechiae, hepatomegaly, jaundice) or abnormal laboratory evaluations (platelets <100 k/mm^3^, ALT/AST above 2.5 upper limit of normal, conjugated bilirubin >1.0 mg/dl). Infants who did not meet the definition for symptomatic at birth and had data within normal limits available for at least two of the categories of birth weight, head circumference and newborn physical exam, were considered to be asymptomatic. Otherwise, symptoms at birth were considered to be unknown. Head imaging was not included in the definitions for symptomatic or asymptomatic at birth, as for many infants, head imaging was not performed until the infants were older. Newborn hearing screening (NBHS) results were also not included in the definitions, as hearing screens cannot confirm the presence of a SNHL and frequently, confirmation of SNHL was not available until the infants were older. Age at first CMV testing was considered as the age when the first CMV test was ordered. Reason for CMV testing was abstracted from the clinician notes at the time CMV testing was ordered. Developmental outcomes were determined by review of clinic records and required at least one note with a documented neurologic exam or description of developmental status. Children were classified as age-appropriate or as having mild to moderate or severe delay (Supplementary Appendix). Children were identified as having SNHL if the pure-tone average (PTA) in decibels hearing level (dBHL) was greater than 30dBHL with bone conduction scores within 10dBHL of air conduction scores. Data were collected for individual ears. Degree of hearing loss, progression of hearing, and alternative data collection strategies is further described in the Supplementary Appendix.

This study was approved by the Seattle Children's Hospital Institutional Review Board (study number 00003616) and the University of Washington Human Subjects Division (STUDY00015697).

### 2.2 Application of different screening strategies

Hearing targeted screening was defined as testing for cCMV based on a failed NBHS, rather than confirmed SNHL, as timely access to diagnostic audiologic evaluation was limited for most infants. Infants without NBHS results available were excluded from this analysis. Expanded targeted screening strategy was based on the expanded targeted early CMV testing protocol described by Suarez et al (22). See Table 2 for a listing of the expanded testing criteria. Mothers were considered to be positive for CMV infection if they were tested in pregnancy and were CMV seropositive. Head imaging results were only included if the imaging had been done within the first 3 weeks after birth and abnormal head size was defined as an occipital frontal circumference (OFC) < 3% rather than the <10% used by Suarez et al (22). Infants were excluded from this analysis if there was insufficient data available from the first three weeks after birth to apply the criteria.

### 2.3 Statistical analysis

Demographic and clinical characteristics of the study population are summarized descriptively using counts with percentages and means with standard deviations or medians with interquartile ranges (IQRs), as applicable. Continuous variables were compared using Mann–Whitney *U* test, and categorical variables were compared using Fisher's exact test. Results were considered statistically significant at a 2-sided *p* < 0.05 and adjusted using the Benjamini-Hochberg method for multiple comparisons. All statistical analyses were performed using R Statistical Software (version 3.6.1; R Foundation for Statistical Computing, Vienna, Austria).

## 3 Results

### 3.1 Patient characteristics and testing details

Five hundred and eighty-nine unique records were obtained for review. One hundred twelve infants with documented cCMV infection were included in this analysis. Reasons for exclusion are detailed in Supplementary Figure S1; Supplementary Table S2. Sixty infants (54%) were diagnosed within the first 21 days of life via urine culture or PCR and/or blood PCR (early diagnosis group EDG) and 52 (46%) were diagnosed beyond this time via neonatal DBS testing (late diagnosis group LDG). Demographic and clinical characteristics, timing and reason for CMV testing are presented in Table 1. Age at first CMV testing was within 21 days for the EDG [median 0 months (IQR 0–0.1)]. For the LDG, the median age at first CMV testing was 11.9 months [IQR 3–41]. Infants with early diagnosis were more likely to be premature (30% vs. 8%) and symptomatic at birth (73% vs. 33%). The majority (77%) of the EDG were tested by the birth hospital with the most common reasons for CMV testing being symptoms noted at birth and having had an abnormal prenatal ultrasound. The majority (67%) of the LDG were tested by otolaryngology/audiology because of SNHL diagnosed beyond a month of life. Notably 17 (33%) of the LDG had symptoms potentially attributable to cCMV at birth that had been missed (7 SGA, 1 microcephaly, 3 both SGA and microcephaly, 2 petechiae, 1 jaundice, 2 thrombocytopenia, 1 hypotonia). For this subgroup of the LDG, the median age at first CMV testing was 18 months (range 1–91 months). Testing was initiated by neurology or genetics after referral for developmental delay for 12 (23%) of the LDG (median age 17.5 months (range 5.4–101.4). Only 3 infants had failed NBHS listed as the sole reason for testing. Fewer of the LDG had eye exams done, but overall, only 5 infants had abnormalities consistent with CMV retinitis and all of these infants also had abnormal CNS imaging even though 2 appeared to be asymptomatic at birth.

**Table 1:** Infant demographics, clinical characteristics and CMV testing details.

	Total cohort *N* (%)	Early diagnosis group (Diagnosed ≤3 weeks of age)	Late diagnosis group (Diagnosed >3 weeks of age)	Adjusted *p* value
	112	60	52	
Gender				
Male	54 (48)	30 (50)	24 (46)	0.85
Female	58 (52)	30 (50)	28 (54)	
Race/ethnicity				
AIAN, NHPI	4 (4)	1 (2)	3 (6)	0.48
Black	8 (7)	3 (5)	5 (10)	0.63
Asian	9 (8)	4 (7)	5 (10)	0.85
White	79 (71)	46 (77)	33 (64)	0.26
Hispanic	18 (16)	6 (10)	12 (23)	0.15
Not stated	14 (13)	7 (12)	7 (13)	0.88
Premature (<37 weeks EGA)	24 (21)	20 (30)	4 (8)	0.002
Presentation at birth				
Symptomatic	61 (55)	44 (73)	17 (33)	<0.001
Failed NBHS	40 (66)	30 (68)	10 (59)	0.25
Passed NBHS	19 (31)	14 (32)	5 (29)	
Unknown	2 (3)		2 (12)	
Asymptomatic	45 (40)	16 (27)	29 (56)	<0.001
Failed NBHS	29 (64)	8 (50)	21 (72)	0.30
Passed NBHS	15 (33)	8 (50)	7 (24)	
Unknown	1 (2)		1 (4)	
Unknown symptoms	6 (5)	0	6 (12)	0.19
Failed NBHS	2 (33)		2 (33)	1
Passed NBHS	3 (50)		3 (50)	
Unknown	1 (17)		1 (17)	
Age at first CMV testing in months -median [IQR]	0.5 [0.00, 7.80]	0 [0.00, 0.10]	11.9 [3.40, 41.15]	<0.001
Department initiating CMV testing				
Birth hospital	47 (42)	46 (77)	1 (2)	<0.001
NICU	7 (6)	6 (10)	1 (2)	
OTO/Audiology	35 (31)	0	35 (67)	
Primary care provider	5 (5)	3 (5)	2 (4)	
Neurology	6 (5)	1 (0.2)	5 (10)	
Genetics	7 (6)	0	7 (13)	
Other^a^	5 (5)	4 (7)	1 (2)	
Reason for testing^b^				
Symptoms at birth^c^	23 (21)	23 (38)	0	<0.001
SGA^d^	14 (13)	14 (23)	0	<0.001
Failed NBHS^e^	6 (5)	5 (8)	1 (2)	0.35
Mother diagnosed with CMV during pregnancy^f^	16 (14)	15 (25)	1 (2)	<0.001
Abnormal prenatal ultrasound^g^	19 (17)	18 (30)	1 (2)	<0.001
SNHL diagnosed beyond a month of life^h^	37 (33.0)	NA	37 (71)	<0.001
Other^i^	19 (17.0)	6 (10)	13 (25)	0.09
Reason not stated	1 (0.9)	1 (2)	0	1
Ophthalmology				
Number evaluated	89 (79)	56 (90)	33 (63)	<0.001
Number (%) abnormal and related to CMV	5 (6)	3 (5)	2 (6)	1
Head imaging^j^				
Number with cranial ultrasound results	61 (55)	49 (82)	12 (23)	<0.001
Number (%) abnormal and related to CMV	34 (55)	29 (59)	5 (45)	0.48
Number with head MRI results	70 (63)	39 (65)	31 (60)	0.72
Number (%) abnormal and related to CMV	58 (83)	32 (82)	26 (87)	1
Number with CT results	3	2	1	
Number (%) abnormal and related to CMV	0 (0)	0 (0)	0 (0)	
Number with no imaging	17 (15)	2 (3)	15 (29)	<0.001

CMV, cytomegalovirus; MRI, magnetic resonance imaging; CT, computed tomography scan; SGA, small for gestational age; NBHS, newborn hearing screen.

^a^ 1 emergency department, 1 pulmonology, 3 infectious disease.

^b^ Could have more than one reason identified.

^c^ Physical exam findings concerning for cCMV—petechiae, jaundice, hepatosplenomegaly, microcephaly.

^d^ 3 with SGA as the only stated reason.

^e^ 3 with failed NBHS as the only reason.

^f^ 8 with maternal CMV positivity as the only stated reason.

^g^ 8 with abnormal prenatal ultrasound as the only stated reason.

^h^ 33 with SNHL diagnosed after 1 month of age as the only stated reason.

^i^ Other category includes only those with alternative reasons for testing.

- Early diagnosis group: 2 with abnormal lab results, 2 with placental pathology concerning for CMV, 1 with pneumonitis, 1 with positive CMV viral culture during a rule-out herpes simplex virus work-up.

- Late diagnosis group: 10 because of developmental delay and abnormal CNS imaging, 1 with placenta positive for CMV, 2 with prolonged thrombocytopenia.

^j^ Could have had more than one imaging modality.

Infants in the LDG were less likely to have head imaging performed: 15/52 (29%) with no imaging done compared to 2/60 (3%) in the EDG without imaging (*p*-value < 0.001) Most infants with head MRI and ultrasound results available had abnormalities consistent with cCMV: 58/70 (83%) and 34/61 (56%) respectively, with no difference between the EDG and LDG. Of the 34 infants with abnormal cranial ultrasounds, 8 (24%) appeared to be asymptomatic at birth. Of the 58 infants with abnormal MRIs, 22 (38%) appeared to be asymptomatic at birth and of these, 14 were in the LDG.

### 3.2 Application of different screening strategies

For this cohort, a universal screening testing strategy could have identified an additional 52 (46%) infants within the entire cohort, potentially leading to 100% of the infants being identified at birth. There were 108 infants with NBHS testing results available. The 4 infants without NBHS data were all in the LDG. If solely a targeted hearing testing strategy was in place, then 71/108 (66%) could have been diagnosed at birth: 38/60 (63%) of the EDG and 33/48 (69%) of the LDG. There were 108 infants with sufficient birth data available to evaluate the expanded targeted early testing approach (22). The 4 with insufficient data were all in the LDG. The number of infants with each specific finding is detailed in Table 2. Applying the expanded targeted early testing strategy to the entire cohort, 99/108 (92%) of the full cohort could have been diagnosed early: 57/60 (95%) of the EDG and 42/48 (88%) of the LDG.

**Table 2:** Number of infants with findings included in the expanded targeted early CMV testing program [ref (22)].

Criteria	Number with finding^a^		
	Total cohort^b^ *N* = 108	Early diagnosis group *N* = 60	Late diagnosis group^b^ *N* = 48
Mother positive for CMV during pregnancy	17	16	1
Abnormal head size^c^	23	19	4
Low birth weight^d^	36	26	10
Intracranial calcifications^e^	11	11	0
Hepatomegaly/splenomegaly	9	9	0
Petechial or “blueberry muffin” rash	18	16	2
Thrombocytopenia^f^	23	20	3
Hepatitis^g^	15	14	1
Abnormal MRI^h^	6	6	0
Unexplained hydrops	0	0	0
Failed NBHS	71	38	33

CMV, cytomegalovirus; MRI, magnetic resonance imaging; NBHS, newborn hearing screen.

^a^ Could have more than one finding.

^b^ Excluding 4 infants without sufficient data in the first three weeks of life to apply criteria.

^c^ Occipital frontal circumference <3rd percentile.

^d^ Weight <10th percentile for gestational age.

^e^ Cranial imaging done prior to 3 weeks of age.

^f^ Platelets <100 k/mm^3^.

^g^ ALT/AST >2.5 upper limit of normal or direct bilirubin >1.0 mg/dl.

^h^ Any of leukomalacia, polymicrogyria, lissencephaly, pachygyria, schizencephaly on an MRI done prior to 3 weeks of age.

### 3.3 Antiviral treatment

Of the 60 infants in the EDG, 41 (68%) were treated with ganciclovir, valganciclovir or both. Of these, 36 (88%) initiated antivirals within the first month of life, 3 were 1 month of age or older and 2 had an unknown start date. All these infants had symptoms consistent with cCMV and/or abnormal head imaging. Of the LDG, 12/52 (23%) were treated with valganciclovir with the median age of initiation of 3.5 months (range 1.25–6). Stated treatment indication in these infants was SNHL for 8, prolonged thrombocytopenia for 2 and microcephaly with SNHL for 2. The most recent American Academy of Pediatrics (AAP) Red Book recommends that valganciclovir should be initiated within the first 13 weeks following birth for infants with moderate to severe symptomatic cCMV (includes evidence of central nervous system involvement) or with isolated cCMV associated SNHL (23). Forty-two of the 52 infants (81%) in the LDG met criteria for treatment with valganciclovir based on the current AAP guidelines: 11 with evidence of central nervous system involvement based on abnormal head imaging and/or microcephaly, 15 with isolated congenital SNHL and 16 with both congenital SNHL and central nervous system involvement. Of these, 9 were tested for CMV within the first 13 weeks following birth, so could potentially have accessed treatment within the window. The remaining 33 infants (63% of the total LDG cohort) may have missed the opportunity for valganciclovir as they were not diagnosed with cCMV until after 13 weeks following birth.

### 3.4 Outcome

#### 3.4.1 Audiology

Of the EDG, 22 passed their newborn hearing screening (NBHS) and 38 did not pass in either one or both ears (Table 1). Of the LDG, 15 passed their newborn hearing screening, 33 did not pass in either one or both ears, and 4 did not have data available.

Diagnostic audiologic evaluations were available for 108 of the children in this study (Table 3). In the EDG, 23 had no documented SNHL and 34 had documented SNHL in one or both ears by their most recent evaluation. Of the 34 with SNHL, 18 showed a progression of SNHL. In the LDG, 5 had no documented SNHL and 46 had documented SNHL in one or both ears by their most recent evaluation. Those with SNHL had a median age at CMV diagnosis of 9.1 months [IQR 3–37], with a median age at first audiologic evaluation of 3.5 months [IQR 1.5–24] (Table 4). Twenty-nine children showed a progression of hearing loss in the LDG. Of those 29, 14 initially passed their NBHS but later demonstrated SNHL with a median age at first SNHL diagnosis of 27 months [IQR 15–42] and a median age at CMV testing of 51 months (IQR 20–70). Additional audiologic outcomes for this cohort are detailed in Tables 3, 4.

**Table 3:** Hearing outcome at longest follow-up.

	Total *N* (%)	Early diagnosis group	Late diagnosis group	Adjusted *p* value
	112	60	52	
Audiology/hearing status				
No documented SNHL	28 (25)	23 (38)	5 (10)	<0.001
SNHL in one or both ears	80 (71)	34 (57)	46 (88)	<0.001
Unilateral SNHL	35 (44)	13 (38)	22 (48)	0.58
Bilateral SNHL	45 (56)	21 (62)	24 (52)	0.58
Progressive hearing loss^a^	47 (42)	18 (30)	29 (56)	0.01
Passed NBHS, then developed SNHL	16 (14)	2 (3)	14 (27)	0.002
Unknown hearing status	4 (4)	3 (5)	1 (2)	0.62

^a^ Progressive hearing loss was considered as a 20dBHL or greater decrease in PTA in at least one ear between evaluations or a passed newborn hearing screening with subsequent audiologic evaluations documenting a SNHL.

**Table 4:** Age at first audiologic evaluation and first CMV testing in the late diagnosis group.

	Late diagnosis group (*N*)	Median age [IQR] at first audiologic evaluation in months	Median age [IQR] at CMV testing in months
Audiology/hearing status			
No documented SNHL	5	29.6 [17.73, 41.57]	22.6 [6.70, 29.00]
SNHL in one or both ears	46	3.5 [1.53, 24.15]	9.1 [3.40, 36.60]
Passed NBHS, then developed SNHL	14	27.2 [14.93, 41.60]	50.9 [19.88, 70.10]
Unknown hearing status	1	1.7	101.4

#### 3.4.2 Development

Developmental outcome information was available for 88 (78.6%) of the children in this cohort with a median age at last assessment of 79 (range 8-201) months. Of these 88, 34 (39%) were age appropriate, 33 (38%) had mild to moderate delays and 21 (24%) had severe developmental delay. Developmental outcomes were similar between the EDG and LDG, however developmental outcome was less likely to be available for the infants in the EDG (28% in the EDG group with unknown developmental outcome vs 13% in the LDG). Of the 54 children with mild to moderate, or severe delays, 29 (54%) were in the LDG with a median age at initial CMV testing of 16.3 (range 1.1-101.4) months. Developmental outcome was available for 12 of the 14 infants in the LDG who were asymptomatic at birth yet had abnormal head imaging. Nine of the 12 had delayed development at longest follow-up: 4 mild to moderate and 5 severe.

## 4 Discussion

In our cohort, almost half of the infants eventually diagnosed with cCMV were missed at birth. Excluding infants with isolated SNHL, over a third of the late diagnosed infants had symptoms consistent with cCMV at birth. This finding is consistent with previous research which suggests that up to 90% of symptomatic cCMV infants may be missed using a clinician-initiated testing model (19). These and other data (20) support that relying on clinicians to appropriately identify symptoms of cCMV at birth in a timely manner is not a sensitive method of diagnosing children with cCMV. Even if all symptomatic infants were tested and diagnosed within the appropriate time period, a large number of infants who lack overt symptoms at birth would go undiagnosed yet remain at risk for development of permanent sequalae.

Quantifying “missed opportunities” for this population is challenging due to the retrospective nature of our data collection. We used median age at CMV testing as the metric to quantify the missed opportunities for treatment, intervention, and monitoring. Children with hearing loss who are identified by 6 months of age and receive interventions have significantly better language development outcomes than those diagnosed with hearing loss after 6 months of age (24). Due to the progressive nature of cCMV related hearing loss, early identification is critical. The American Academy of Audiology recommended guidelines for all children diagnosed with cCMV, regardless of their hearing status, suggest frequent monitoring over the first few years of life (25). This audiologic monitoring schedule is necessary due to the progressive and unpredictable nature of CMV related hearing loss (12, 26, 27). Appropriate audibility is paramount to the development of spoken speech and language. In our cohort, the median age at the first audiologic assessment for children in the LDG who passed their newborn hearing screening but later developed a SNHL was over 2 years. Had these children been diagnosed with CMV at birth and received appropriate audiologic monitoring, they would have been evaluated eight times prior to their second birthday. This may have provided opportunities for earlier SNHL diagnosis and audiologic intervention. Additionally, these children were not diagnosed with cCMV for a further two years. This lack of knowledge about their hearing loss etiology and other delays could have further impeded access to appropriately timed interventions.

Our finding that a significant percent of infants with cCMV had abnormal findings on head imaging and/or some degree of developmental delay even though they appeared to be asymptomatic at birth is consistent with data from other cohorts (1, 7, 28–30). While not all infants with abnormalities on head imaging, particularly those with isolated white matter changes or lenticulostriate vasculopathy, are at risk for abnormal development (31, 32), limiting CMV testing to those infants with classic cCMV symptoms, risks a delayed diagnosis for many infants at risk for CMV-related developmental abnormalities. Studies investigating the etiology for children with developmental abnormalities such as cerebral palsy identified cCMV based on DBS or umbilical blood testing in 10%–30% (33, 34). Similarly, in our cohort, 23% of the infants in the LDG were belated tested for cCMV because of otherwise unexplained developmental delays.

Infants with a delayed cCMV diagnosis also may miss the window of opportunity for antiviral treatment. Infants with symptomatic cCMV initiated on valganciclovir within the first month of life and continued for 6 months have been shown to have improved hearing and developmental outcomes (35). Previous recommendations for valganciclovir limited treatment to symptomatic infants who could be initiated on valganciclovir within one month of age (36). A recently published non-randomized controlled trial compared hearing and developmental outcomes in 36 infants with cCMV-related SNHL treated with six weeks of valganciclovir vs. no treatment. In this trial, in which infants were <13 weeks of age at enrollment, treated infants had improved hearing outcomes, with no difference in developmental outcome (37). Based on this study, the AAP broadened the recommendation for treatment with valganciclovir to include 6 months of valganciclovir for symptomatic infants with initiation within the first 13 weeks following birth and 6 weeks of valganciclovir for infants with isolated SNHL also with initiation within the first 13 weeks following birth (23). Even with the extended time frame allowed for initiation of valganciclovir, almost two-thirds of infants in the LDG of our cohort who met criteria for valganciclovir treatment would have missed the opportunity as they were diagnosed after 13 weeks following birth.

Our data show that any formal screening protocol (universal, hearing targeted, or expanded targeted) would result in earlier diagnosis for most infants. Using a referred newborn hearing screening as the trigger to test for CMV, also known as a hearing targeted screening, is a commonly adopted practice throughout clinics and hospitals and mandated in several states within the United States (18, 38). While many infants with cCMV related hearing loss will be identified through a hearing targeted CMV screening protocol, approximately 40% with CMV related SNHL in the neonatal period will be missed as will those who develop SNHL later in childhood (39). Consistent with the above, applying only a hearing targeted testing strategy to our cohort would have identified over 60% of the infants. In actuality, a strictly hearing targeted strategy for testing is unlikely as many infants with classic cCMV symptoms will be tested regardless of NBHS results. For our cohort, assuming the EDG group would still have been diagnosed early, adding a hearing targeted testing strategy could have identified an additional third of our total cohort.

Using an expanded targeted CMV testing protocol which includes additional risk factors such as persistent thrombocytopenia and maternal CMV infection may improve detection rates (22). This is confirmed by our cohort as nearly all the infants of both subgroups (EDG and LDG) could have been diagnosed within the appropriate time frame if expanded targeted CMV testing had been in place.

This study has several important limitations. Significantly, as this was a retrospective chart review the data was limited to what was available in the medical record. There was no standard for following infants and children diagnosed with cCMV and those with more severe outcomes generally had longer and more detailed follow-up data available. In particular, infants with earlier birth dates were biased towards worse outcomes as only those with continued medical issues would have been included in the CDR database. The LDG was biased towards those infants identified due to a SNHL and no data is available for potential infants with asymptomatic cCMV who escaped permanent sequelae or for those who failed to be diagnosed. As CMV testing on neonatal DBS specimens was not available prior to 2008, the median age at diagnosis was elevated in the early years. In addition, as on average it takes about a month to get the DBS CMV testing results, infants in the LDG were even older than our data suggests by the time a cCMV diagnosis was confirmed. Due to these limitations and based on the design of this study, epidemiological questions cannot be addressed.

Few children had formal developmental testing, therefore the determination of developmental outcome was based on physician assessment, physical exam and parental report, thus no attempt was made to distinguish between mild and moderate delay.

## 5 Conclusions

This study highlights the impact of cCMV infection on infants and children and the need to confirm a diagnosis as soon as possible. The current lack of formal universally adopted recommendations for testing for cCMV at birth leads to delayed diagnosis for many infants. This delay results in missed opportunities for monitoring, intervention, and treatment.

## Data Availability

The original contributions presented in the study are included in the article/Supplementary Material, further inquiries can be directed to the corresponding author.
