# Supplementary material for: Timing of congenital cytomegalovirus diagnosis and missed opportunities
Source: Front Pediatr. 2025 Feb 4;13:1475121. doi: 10.3389/fped.2025.1475121 (PMC11832646; doi:10.3389/fped.2025.1475121)
Supplement: Supplementary file 1 [file Image1.pdf]

Figure 1: Process for record identification and review to obtain final cohort of infants with documented cCMV infection.

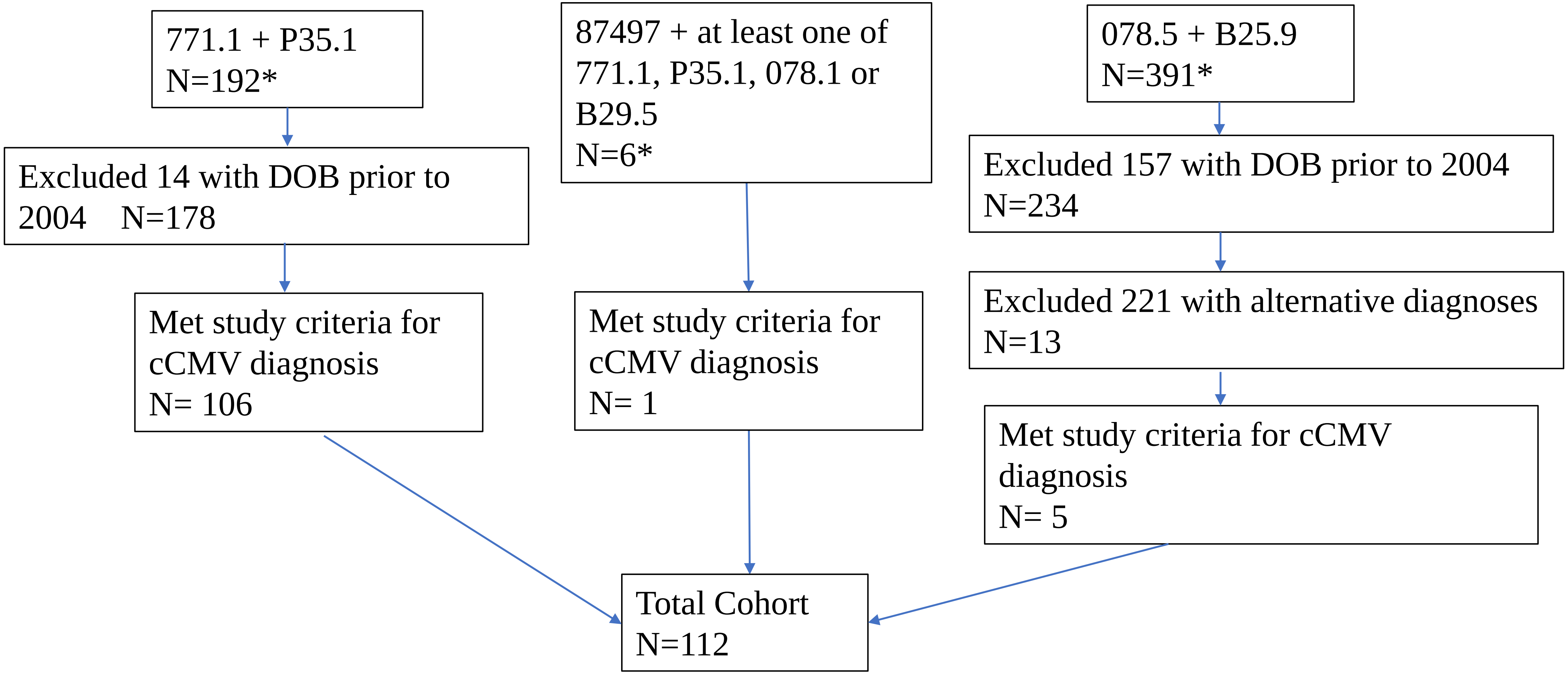

* Excluding duplicates
