# Supplementary material for: Timing of congenital cytomegalovirus diagnosis and missed opportunities
Source: Front Pediatr. 2025 Feb 4;13:1475121. doi: 10.3389/fped.2025.1475121 (PMC11832646; doi:10.3389/fped.2025.1475121)
Supplement: Supplementary file 2 [file Table1.docx]

**Table 1: Search methods**

| **ICD code** | **ICD version** | **Diagnosis** |
| --- | --- | --- |
| 771.1 | ICD-9 | Congenital cytomegalovirus infection |
| P35.1 | ICD-10 | Congenital cytomegalovirus infection |
| 078.5 | ICD-9 | Cytomegaloviral disease |
| B25.9 | ICD-10 | Cytomegaloviral disease |
| **CPT code** | **Lab code** | **Test name** |
| 87497 | CMVBSQNT | CMV by PCR, newborn blood spot |

**Table 2: Reason for exclusion of records after additional clinical review for documentation of congenital CMV diagnosis**

| **Exclusion categories** | **Number excluded** |
| --- | --- |
| Virologic testing^a^ results available within 21 days of age and negative for CMV | 27 |
| No virologic testing^a^ results available within 21 days of age | 46 |
| Transferred to SCH with cCMV diagnosis but insufficient data available for confirmation of diagnosis and data abstraction | 12 |

a including DBS test results

**Classification of neurologic impairment**

**Age appropriate –** no mention of neurologic or developmental impairment, in a regular age-appropriate classroom or preschool

**Mild to moderate delay –**notes indicate some degree of motor or cognitive delays**,** for children with a cerebral palsy diagnosis, Gross Motor Classification System^1^ (GMFCS I-III)

**Severe delay –**global developmental delay with a diagnosis of cerebral palsy GMFCS score IV-V

**Audiologic methods and classification**

Pure-tone average (PTA) in decibels hearing level (dBHL) was calculated for each ear as the average of thresholds obtained at 500Hz, 1000Hz, and 2000Hz. If a threshold was not available at one or more of the frequencies, then an average was taken for two thresholds or a single threshold was used as the PTA.

Normal hearing (-10dBHL to 20dBHL), mild (25dBHL to 40dBHL), moderate (45dBHL to 55dBHL), moderately severe (60dBHL to 65dBHL), severe (70dBHL to 85dBHL), and profound (90dBHL and greater).

Degree of hearing and/or PTA were collected for the following time periods: earliest audiologic data available, first audiologic data at Seattle Children’s Hospital, data collected between the age of 18 months and 30 months, and the most recent evaluation. For some subjects one evaluation may have been considered for several categories.

Once SNHL was established in an ear, it was considered SNHL for all subsequent tests. When the PTA was not available, the degree of hearing loss was recorded.

Progressive hearing loss was considered as a 20dBHL or greater increase in PTA in at least one ear between evaluations or a passed NBHS with subsequent audiologic evaluations documenting a SNHL. For patients identified with progression, a further analysis of audiologic records was conducted. Each available audiogram or report was analyzed by MRB. Change in hearing due to abnormal tympanometry or unreliable testing was not included as a progressive hearing loss. Once a subject received a cochlear implant, the implanted ear was considered “no response” and classified as profound.

Alternative Data Collection Strategies: If individual ear data was unavailable due to soundfield testing, it was recorded as such.

**References**

1. CanChild Resources GMFCS-E&R. Retrieved April 17, 2024 from https://canchild.ca/en/resources/42-gross-motor-function-classification-system-expanded-revised-gmfcs-e-r.
